# Activation function 1 of progesterone receptor is required for progesterone antagonism of oestrogen action in the uterus

**DOI:** 10.1186/s12915-022-01410-3

**Published:** 2022-10-05

**Authors:** Shi Hao Lee, Chew Leng Lim, Wei Shen, Samuel Ming Xuan Tan, Amanda Rui En Woo, Yeannie H. Y. Yap, Caitlyn Ang Su Sian, Wilson Wen Bin Goh, Wei-Ping Yu, Li Li, Valerie C. L. Lin

**Affiliations:** 1 School of Biological Sciences, Nanyang Technological University, Singapore, 637551 Singapore; 2 College of Informatics, Huazhong Agricultural University, Wuhan, China; 3 Present Address: Department of Oral Biology and Biomedical Sciences, Faculty of Dentistry, MAHSA University, Bandar Saujana Putra, 42610 Jenjarom, Selangor Malaysia; 4 Animal Gene Editing Laboratory (AGEL), Biological Resource Centre, Agency for Science, Technology and Research (A*STAR), 61 Biopolis Drive, Proteos, Singapore, 138673 Singapore; 5 Institute of Molecular and Cell Biology, Agency for Science, Technology and Research (A*STAR), 61 Biopolis Drive, Proteos, Singapore, 138673 Singapore

**Keywords:** PGR, Activation function 1, Endometrium, ESR1, Genomic antagonism

## Abstract

**Background:**

Progesterone receptor (PGR) is a master regulator of uterine function through antagonistic and synergistic interplays with oestrogen receptors. PGR action is primarily mediated by activation functions AF1 and AF2, but their physiological significance is unknown.

**Results:**

We report the first study of AF1 function in mice. The AF1 mutant mice are infertile with impaired implantation and decidualization. This is associated with a delay in the cessation of epithelial proliferation and in the initiation of stromal proliferation at preimplantation. Despite tissue selective effect on PGR target genes, AF1 mutations caused global loss of the antioestrogenic activity of progesterone in both pregnant and ovariectomized models. Importantly, the study provides evidence that PGR can exert an antioestrogenic effect by genomic inhibition of *Esr1* and *Greb1* expression. ChIP-Seq data mining reveals intermingled PGR and ESR1 binding on *Esr1* and *Greb1* gene enhancers. Chromatin conformation analysis shows reduced interactions in these genes’ loci in the mutant, coinciding with their upregulations.

**Conclusion:**

AF1 mediates genomic inhibition of ESR1 action globally whilst it also has tissue-selective effect on PGR target genes.

**Supplementary Information:**

The online version contains supplementary material available at 10.1186/s12915-022-01410-3.

## Background

Progesterone receptor (PGR) is a transcription factor of the nuclear receptor superfamily. The transcriptional activity of nuclear receptors is primarily mediated by activation functions AF1 and AF2. AF1 is in the intrinsically disordered N terminal domain (NTD), whereas AF2 is in the structurally conserved ligand binding domain (LBD). AF1 and AF2 provide an interface for the recruitment of transcription coregulators that modify chromatin and facilitate the assembly of general transcription machinery [1, 2]. It is established that AF2 is critical for ligand-dependent gene regulation, whereby ligand binding induces the formation of coregulator interaction surface. Although AF1 was initially thought to mediate ligand-independent activity [3], there is increasing evidence to suggest that it also plays important roles in ligand-dependent and gene- and tissue-specific activity [2]. However, the understanding of the AF1 function of steroid receptors in vivo is limited due to the lack of a suitable genetic model. So far, the only reported genetic model for the study of AF1 is the mouse model with ESR1 AF1 or AF2 domain deletion. Studies of the model have provided valuable insight into the function significance of ESR1 AF1. The AF1-deleted mice exhibit various developmental defects in the target organs including the uterus, mammary gland, bone, and cardiovascular system [4–6]. Notably, ESR1 AF-1 is necessary and sufficient for the proliferation of uterine epithelial cells [6].

Our previous proteomic analysis identified three critical amino acids (K464, K481, R492) that are monomethylated in human PGR AF1 [7, 8]. Combined mutations of these residues to phenylalanine (F), a putative methylation mimic, resulted in a loss of PGR activity by ~ 80% in gene reporter assays [8]. The 3 residues are evolutionally conserved and correspond to K461, K478, and R489 in mice (Additional file 1: Fig. S1A). To clarify PR AF1 function in vivo, a mouse line with triple mutations (K461F, K478F, R489F) was generated by CRISPR-Cas9-mediated gene editing. The AF1 mutant (AF1_FFF) mouse is infertile with implantation and decidualization defect. The present study focuses on the understanding of PGR AF1 function in the uterus.

The uterus consists of an inner layer of endometrium, a middle layer of myometrium, and the outer perimetrium. The endometrium of a single layer of luminal epithelium and the underlying stroma. The endometrial glands in the stroma derive from the invagination of the luminal epithelium [9]. Progesterone and oestrogen are master regulators of endometrial function through coordinated antagonistic and synergistic interplays. This interplay is exemplified in uterine preparation for embryo implantation after fertilization. In mice in the first 1–2 days of gestation, rising levels of oestrogen stimulate luminal and glandular epithelial proliferation and target gene expression [10]. From gestation day (GD) 2.5, increasing levels of epithelial PGR and circulating progesterone inhibit oestrogen action to facilitate endometrium receptivity of embryo implantation on GD4.5. The antagonistic effect of progesterone on oestrogen is also an important for endometrial homeostasis as oestrogen hypersensitivity due to impaired progesterone signalling is a common aetiology of infertility, endometriosis and endometrial cancer [11–13]. An in-depth knowledge of the mechanisms of progesterone antagonism of oestrogen action is therefore important for the clinical management of endometrial diseases. The present study indicates that the activity of PGR AF1 is critical for the antioestrogenic effect of progesterone. Importantly, loss of the antioestrogenic activity in the AF1 mutant is not associated with significant downregulation of canonical IHH-PCTH1-NR2F2 or HAND2-IGFs signalling. Instead, the study presents evidence for a genomic mechanism of antagonism, in which PGR is involved in ESR1-regulated gene expression through chromatin interactions in an AF1-dependent manner.

## Results

### AF1_FFF mice exhibit defect in embryo implantation and uterine decidualization

**Fig. 1:**
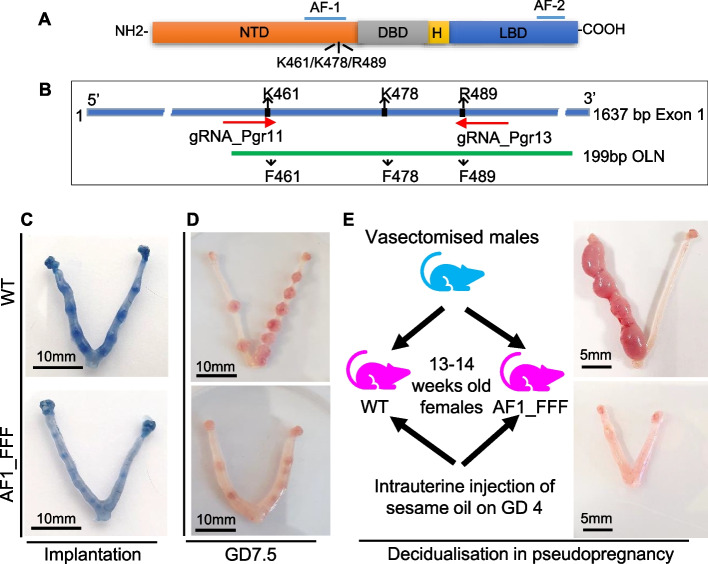
AF1_FFF mice exhibit defect in embryo implantation and uterine decidualization. **A** Domain structure of PGR and the 3 critical amino acids of AF1 in the mouse PGR. **B** CRISPR-Cas9-mediated gene editing strategy for generating the mouse line with triple mutant Pgr. The long blue bar indicates Exon 1 of the Pgr gene. The AF1_FFF mouse was generated by pronuclei injection of Cas9 mRNA, 2 Pgr gRNAs, and the homologous single-stranded oligonucleotide (OLN) with the mutations. **C** AF1_FFF mice exhibit impaired embryo implantation on GD4.5. **D** The AF1_FFF foetuses develop poorly on GD7.5. **E** The AF1_FFF uterus shows the failure of decidualization in pseudopregnant mice

Lysine K461, K478, and arginine R489 of the mouse PGR were mutated to phenylalanine (F) in C57BL/6J mice to generate PGR with K461F_K478F_R489F mutations by CRISPR/Cas9-mediated gene editing (Fig. 1B). The genotyping strategy is shown in Additional file 1: Fig. S1A. Levels of PGR protein in the uterus of the triple mutant (AF1_FFF) are similar to that in the wild type (WT) mice after treatment with 17β-estradiol benzoate (EB) (Additional file 1: Fig. S1B). There were two considerations for measuring uterine PGR expression in oestrogen-treated ovariectomized mice. First, the PGR levels would be quite variable in virgin mice depending on the stage of the oestrous cycle. Second, since PGR is oestrogen-inducible, the ovariectomized mice provide a comparable hormonal background to gauge whether PGR regulation and protein stability are altered in the mutant. Based on the analysis in breast cancer cells MCF-7, AF1_FFF mutation did not affect the nuclear localization of PGR (Additional file 1: Fig. S2).

The homozygous AF1_FFF female mice did not give any live birth and are therefore 100% infertile. However, bloody vaginal discharges were spotted in some of the AF1_FFF mice on 9–12 days post-coitum, indicating miscarriages. This suggests that ovulation, fertilization, and implantation had occurred in some of the mutant mice. Indeed, embryo implantation assay using Evans Blue showed signs of implantation in some of the AF1_FFF mice at gestation day 4.5 (GD4.5), but the implantation bands in the mutant mouse were visibly weaker than the WT mouse (Fig. 1C). Furthermore, live foetuses were seen in some of the AF1_FFF mice on GD 7.5, albeit severely underdeveloped (Fig. 1D). These foetuses were invariably aborted because none of the AF1_FFF female mice gave birth to live pups (Additional file 1: Fig. S1C). Artificial decidualization assays using pseudopregnant mice showed decidualization failure in AF1_FFF mice (Fig. 1E). Together, these observations suggest that PGR AF1 is required for embryo implantation, decidualization, and maintenance of pregnancy. On the other hand, the genotype of the embryo did not affect its development into a pup in Het mothers because Het × Het breeding gave rise to the expected genotypic ratio of 1:2:1 (21.7:53.1:25.2). Homo_male × Het_female breeding also produced normal litter size with healthy homo pups. Hence, the mutation did not affect embryo development intrinsically. The impairment of embryo implantation in Homo AF1_FFF female mice is due to defective PGR signalling on the uterus.

### AF1_FFF uterus on GD3 displays transcriptome of heightened oestrogenic response

Since PGR is a transcription factor, RNA-Seq analysis was conducted to understand the transcriptional defect of the AF1_FFF mutant in the uterus of early pregnancy on GD3 and GD4.5. The morning when the mating plug was found was designated as GD0.5. The mice in the late afternoon of the same day were designated to be on GD1. GD3 is chosen as the time point for the evaluation of PGR activity in the epithelium because it is the time when the endometrial epithelial PGR expression is predominant [10, 14]. GD3 is also the time when the plasma concentration of progesterone reaches near peak level [15]. GD4.5 is the time point of the initiation of embryo implantation and endometrial decidualization. Pgr expression on GD 4.5 is absent in the luminal epithelium but abundant in the endometrial stroma. GD4.5 is therefore a good time point for evaluating the effect of AF1 on stroma cells.

PGR is important for ovarian development and ovulation [16, 17]. Whilst we did not characterize the effect of AF1 mutations on the development of the ovary, we did notice that the frequency of copulatory plugs in AF1_FFF mice was lower than that in the WT mice in timed mating. Since mice are only receptive to mating during the estrus, this suggests abnormalities in ovarian development and the oestrous cycle. Nonetheless, mice included in the study on GD3 and GD4.5 all had mating plugs and were therefore assumed to be in the estrus. Based on the observation in 4.5 days post-coitum mice, more than 90% of the mice with mating plugs had embryos in both genotypes. It can be deduced that the GD3 mice with mating plugs also had embryos at a rate similar to that of GD4.5 mice. It is reasonable to assume therefore that the mutant mice used in this study underwent the ovarian cycle at the time of mating. We also measured serum levels of progesterone on GD3 and 4.5 by PRE-Luc assay based on standard progesterone concentrations in the standard curve. There was no statistically significant difference in the concentration of progesterone between the genotypes on GD3 and GD4.5 that had mating plugs (Additional file 1: Fig. S3). Although the serum concentration of progesterone on GD3 appears lower in the AF1_FFF mutant (albeit statistically insignificant), a study of the experimental cycle in healthy 18- to 35-year-old women reported that a 4-fold difference in progesterone concentration (15–25 ng/ml vs 4–6 ng/ml) did not significantly affect endometrial structure and function based on histological endometrial dating, immunohistochemical, immunoblotting, and RT-qPCR of functional markers [18].

**Fig. 2:**
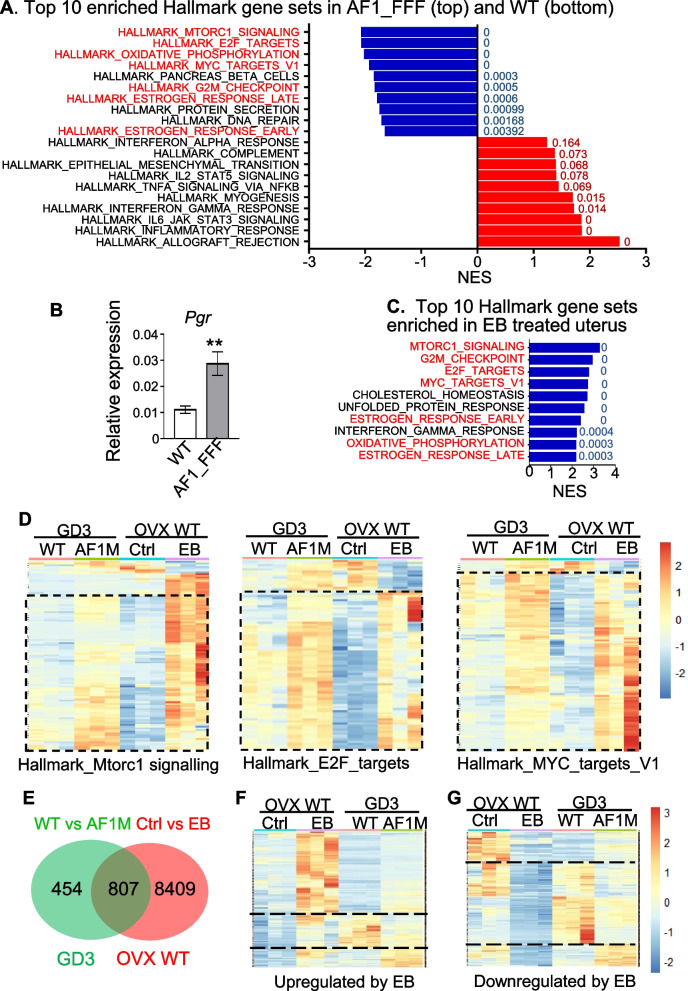
The GD3 AF1_FFF uterus displays transcriptome of heightened oestrogenic response. **A** Top 10 hallmark gene sets enriched in the GD3 AF1_FFF (upper 10 hallmarks in blue) and WT uterus (lower 10 hallmarks in red). **B** *Pgr* expression by RT-qPCR is upregulated in AF1_FFF uterus on GD3. The results are expressed as mean ± SEM (*n* = 6, **p < 0.01). **C** Top 10 hallmark gene sets enriched in EB-treated uterus of OVX mice. Seven of the 10 hallmark gene sets enriched in the GD3 AF1_FFF uterus are enriched in EB-treated uterus (in red). The numbers next to the bars in **A** and **C** are FDR *q* values. NES, normalized enrichment score. **D** Heatmaps indicate that 80–90% (in the dotted rectangles) of EB-upregulated genes in Mtorc1 signalling, E2F targets, and MYC targets gene sets were also upregulated in the AF1_FFF uterus on GD3. The heatmaps are based on core enriched gene sets positively regulated by EB compared to Ctrl in the OVX WT uterus. **E** A total of 807 of the 1261 dysregulated genes in the AF1_FFF uterus are EB regulated in OVX mice. **F**, **G**, Heatmaps indicate that 60–70% of the up- and downregulated genes by EB are also correspondingly up- or downregulated in the AF1_FFF (AF1M) uterus on GD3. The genes above or below the dotted lines in **F** are upregulated by EB in the OVX mouse uterus and AF1_FFF uterus. The genes between the dotted lines in **G** are downregulated by EB in the OVX mouse uterus and in the AF1_FFF uterus. The RNA-Seq analysis was conducted in triplicates. All gene sets used in heatmap have padj < 0.05

The GSEA analysis in this study was conducted by comparing the data of the WT with that of the AF1 mutant. Gene sets with positive NES indicate their over-representation (upregulation) in WT samples. Gene sets with negative NES indicate their downregulation in WT samples and hence over-representation in the AF1_FFF sample. Gene set enrichment analysis (GSEA) of RNA-Seq data revealed that estrogen_response_late and estrogen_response_early were also among the top 10 hallmark gene sets enriched in AF1_FFF samples (Fig. 2A). This suggests hyper oestrogenic response in the AF1 mutant uterus due to the loss of antioestrogenic activity of PGR. This is not due to the decrease in *Pgr* expression. In fact, as an oestrogen target gene, *Pgr* was also significantly upregulated in the AF1-FFF uterus on GD3 (Fig. 2B). To verify that oestrogen-regulated genes are upregulated in the GD3 uterus, we compared dysregulated genes and pathways of GD3 AF1_FFF uterus with that of oestrogen regulated genes in ovariectomized WT mice treated with control vehicle (Ctrl) or estradiol benzoate (EB). Remarkably, 7 of the top 10 hallmark gene sets enriched in AF1_FFF samples were also among the top 10 gene sets enriched in EB-treated samples (Fig. 2A, C, gene sets highlighted in red). Gene sets for cell proliferation, Mtorc1_signaling, E2F_targets, and MYC_targets_V1 were among the top gene sets enriched in both AF1_FFF and EB-treated samples. These are known oestrogen-regulated genes important for cell proliferation [19–22]. Heatmaps of the core enriched genes in these gene sets showed that approximately 80–90% of the genes upregulated by EB in WT uteri were also correspondingly upregulated in AF1_FFF uteri (Fig. 2D). Furthermore, of the 1261 dysregulated genes in the GD3 AF1_FFF uterus (Additional file 2), 807 genes were EB-regulated genes in the WT uterus (Fig. 2E, Additional file 3). Heatmaps of the 807 genes show that ~ 70% of the genes upregulated by EB were also upregulated in the AF1_FFF uterus (Fig. 2F), and ~ 60% of the EB downregulated genes were also downregulated in the AF1_FFF uterus (Fig. 2G). Collectively, the AF1_FFF uterus on GD3 displays a genome-wide hyper oestrogenic response.

### AF1_FFF uterus on GD3 shows delay in cessation of epithelial proliferation

**Fig. 3:**
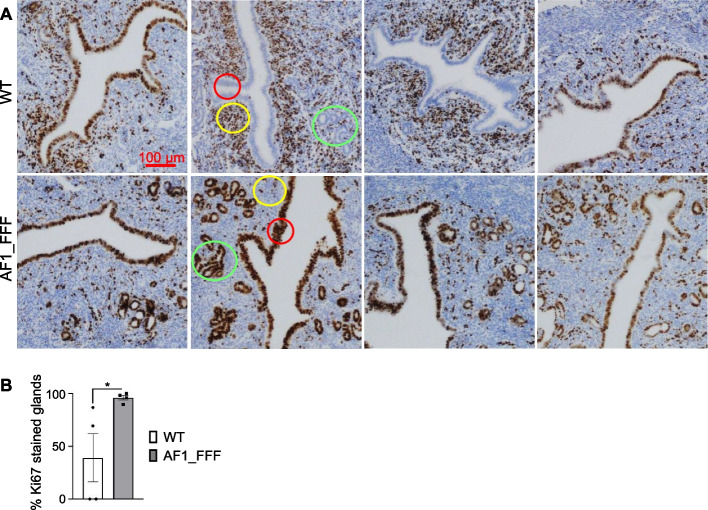
The GD3 AF1_FFF uterus displays delay in cessation of epithelial cell proliferation. **A** Four samples from each genotype were stained with Ki67 antibody. Two of the WT samples are negative for Ki67 in the luminal epithelia (in red circles); in contrast, all 4 AF1_FFF samples are intensely Ki67-positive. The glandular staining of Ki67 in all 4 WT samples is largely absent but is strongly positive in the mutant (in green circles). The stromal cell proliferation in the WT uterus has notably initiated, but it was scarce in the mutant (in yellow circles). **B** The average percentage of Ki67-positive endometrial glands in AF1_FFF uteri is significantly higher than that in the WT uteri (*p* < 0.05, *n* = 4)

At GD 2–3, rising levels of plasma progesterone and epithelial PGR inhibit oestrogen-induced epithelial cell proliferation to facilitate embryo attachment and invasion. The upregulation of Mtorc1_signaling, E2F_targets, and MYC_targets_V1 gene sets in the GD3 AF1_FFF uterus suggests a loss of the anti-proliferative effect of progesterone. Indeed, immunostaining of cell proliferation marker Ki67 showed that luminal epithelial proliferation has ceased in 2 of the 4 WT samples on GD3 (Fig. 3A). Furthermore, glandular epithelial cell proliferation was largely absent in WT samples. In contrast, all four AF1_FFF samples were still intensely positive for Ki67 in the luminal and glandular epithelium, and it is significantly different from the WT samples (Fig. 3B, *p* < 0.05). The observation suggests a delayed cessation of epithelial proliferation in the AF1_FFF uterus. On the other hand, the stromal cell proliferation has initiated abundantly in the WT samples as compared to scarce stromal cell proliferation in AF1_FFF samples (Fig. 3).

### AF1_FFF uterus on GD4.5 also displays hyper oestrogenic transcriptome

**Fig. 4:**
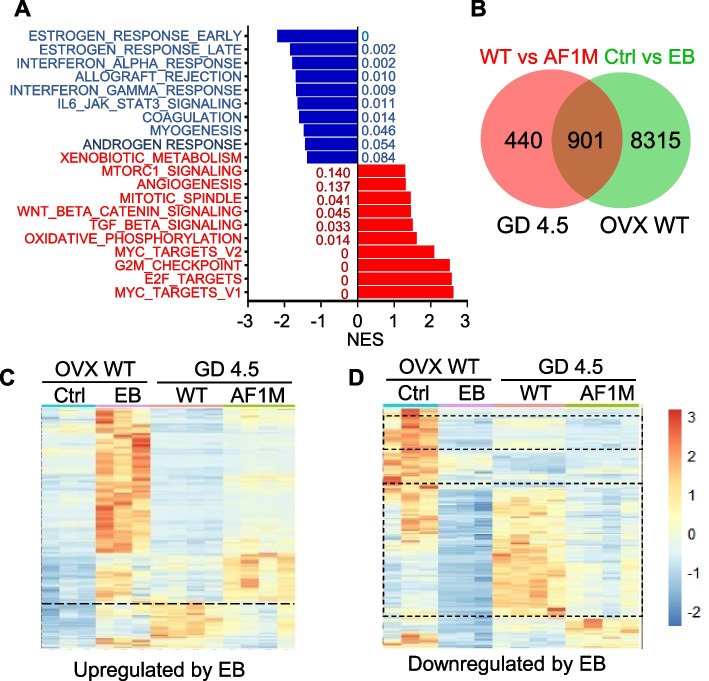
The GD4.5 AF1_FFF uterus also exhibits transcriptome of heightened oestrogenic response. **A** Top 10 hallmark gene sets enriched in the AF1_FFF (blue) and WT (red bars) uteri. The numbers next to the bars are FDR *q* values for each gene set. NES, normalized enrichment score. **B** Venn diagrams indicating the number of genes commonly regulated by EB in OVX mice and dysregulated in the AF1_FFF (AF1M) uterus (padj < 0.05). **C**, **D** Heatmaps indicating that approximately 75% up- and downregulated genes by EB (padj < 0.05) are also correspondingly up- or downregulated in the AF1_FFF (AF1M) on GD4.5. In **C**, genes above the dotted line are commonly upregulated. In **D**, genes within the dotted rectangle are commonly downregulated (*n* = 3 for OVX WT mice; *n* = 4 for GD4.5 mice)

The uterus on GD4.5 is characterized by the loss of epithelial PGR and abundant expression of stromal PGR [10, 23]. Interestingly, estrogen_response_early and estrogen_response_late are the topmost hallmark gene sets enriched in the GD4.5 AF1_FFF uterus compared to the WT (Fig. 4A). DESeq analysis revealed 1341 significantly dysregulated genes (Additional file 4). A total of 901 (67%) of the 1341 dysregulated genes in the AF1_FFF uterus were EB regulated in the OVX WT uterus (Fig. 4B; Additional file 5). Furthermore, approximately 75% of the genes upregulated or downregulated by EB in the WT uterus were also correspondingly up- or downregulated in the AF1_FFF uterus (Fig. 4C, D). Although the magnitudes of the dysregulations in the AF1 mutant may seem less than that regulated by EB, the differences for all genes are statistically significant (padj < 0.05). Hence, AF1-dependent antioestrogenic activity is also a dominant feature in the GD4.5 uterus. However, there is less than 20% overlap in the dysregulated oestrogen target genes between the GD3 and GD4.5 AF1_FFF uterus (Additional file 6). This is likely due to the differential expression of the epithelial and stromal PGR and differential gene regulation by oestrogen between the two developmental states.

### GD4.5 AF1_FFF uterus exhibits downregulation of E2F targets and stromal proliferation

**Fig. 5:**
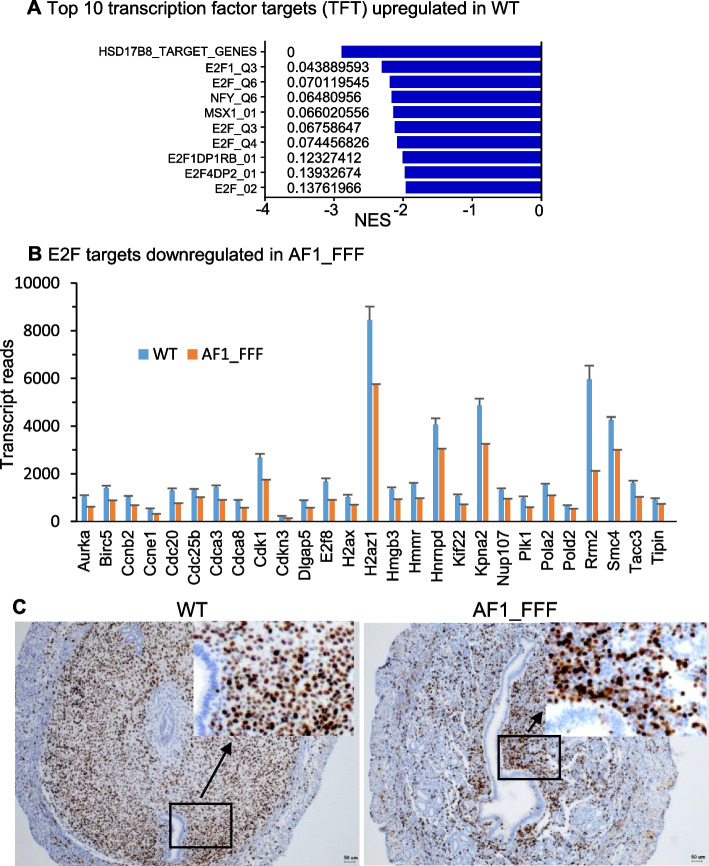
Downregulation of E2F targets in the AF1_FFF uterus on GD4.5 is associated with reduced stromal cell proliferation. **A** Seven of the top 10 TFTs enriched in the WT are E2F targets (FDR < 0.25). **B** The core enriched E2F target genes were all downregulated in the AF1_FFF uterus. The graph is plotted with transcript reads of each gene (mean ± SEM, *n* = 4, padj < 0.05). **C** The AF1_FFF stromal tissue shows fewer Ki67-positive cells than the WT. The images are representative of four samples in each genotype. The region in the black rectangle is enlarged to show clearer Ki67 staining in the insets

In contrast to the upregulation of cell proliferation gene sets, MYC_targets, E2F_targets, and G2M checkpoint in GD3 AF1_FFF mutants, these same gene sets were the topmost enriched hallmark gene sets in the WT uterus on GD4.5 (Fig. 4A). This means downregulation of cell proliferation genes in the mutant at the stage when stromal cell proliferation is prevalent. Interestingly, GSEA of all transcription factor targets (TFT) showed that 7 of the top 10 enriched TFT were E2F targets (Fig. 5A). The core enriched genes in the hallmark E2F_targets were all significantly downregulated in the mutant (padj < 0.05, Fig. 5B). The E2F family of transcription factors plays a key role in cell cycle progression. The downregulation of these genes was expectedly associated with reduced stromal cell proliferation in the AF1_FFF uterus on GD4.5 (Fig. 5C). Together, the data shows that AF1 is critical for PGR to stimulate stromal cell proliferation, and the E2F family of transcription factors is among the key downstream mediators of progesterone-induced stromal cell proliferation.

### AF1 is critical for antagonistic and synergistic activity of progesterone on oestrogen action

#### AF1 is required for progesterone to inhibit oestrogen-induced uterine growth

**Fig. 6:**
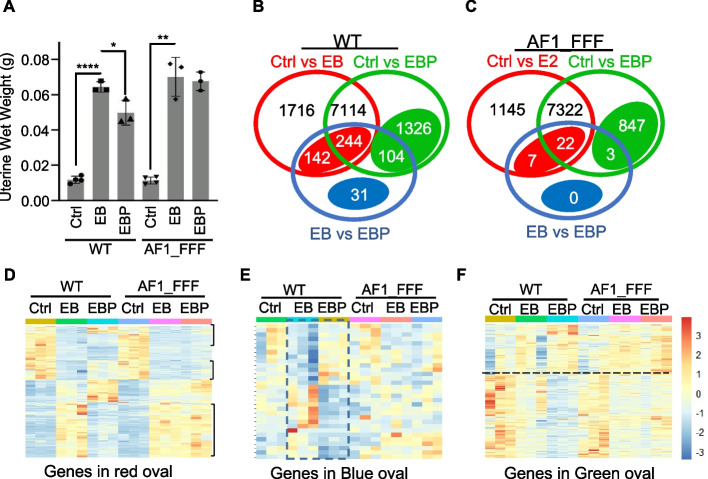
AF1 mediates the antagonistic and synergistic effects of progesterone on oestrogen action in OVX mice. **A** Progesterone failed to inhibit EB-induced uterine wet weight gain in AF1_FFF mice (*n* = 4 in control, *n* = 3 in EB- and EBP-treated samples. All data points are plotted, *p < 0.05). **B**, **C** Venn diagrams show gene regulation by EB and EBP in the WT and AF1_FFF uterus, respectively (padj < 0.05). The number in red ovals indicates genes commonly regulated between Ctrl and EB and between EB and EBP. The number in green ovals indicates genes significantly regulated by EBP compared to Ctrl but not by EB. The number in blue ovals indicates genes differentially regulated between EB and EBP, but not between Ctrl and EB, or between Ctrl and EBP. **D** The heatmap of the 386 genes in the red oval of **B**. Genes indicated by open brackets are oppositely upregulated by EB and progesterone; genes outside the brackets appear synergistically up- or downregulated by EB and progesterone. **E** The heatmap based on the transcripts of the 31 genes in the blue oval of **B**. Genes in the dotted rectangle appear oppositely regulated by EB and progesterone. **F** The heatmap based on the transcripts of the 1430 genes in the green oval of **B**. Genes above the dotted line are upregulated between Ctrl and EBP; genes below are downregulated between Ctrl and EBP

To further elucidate the extent of AF1 involvement in the interplay between progesterone and oestrogen, we evaluated the effect of AF1 mutations on uterine response to treatment with EB or EB plus progesterone (EBP) in OVX mice. Expectedly, EB-induced uterine wet weight gain in WT OVX mice and the addition of progesterone to EB (EBP) significantly inhibited the effect of EB (Fig. 6A). The observation is consistent with gene expression data that EB positively regulates cell proliferation gene sets, G2M checkpoint, E2F targets and MYC targets and about 60% of the G2M checkpoint and E2F targets positively regulated by EB were inhibited by progesterone (Additional file 1: Fig. S4, Additional file 7). However, progesterone failed to inhibit EB-induced uterine weight gain in the AF1_FFF uterus (Fig. 6A). Accordingly, progesterone did not inhibit oestrogen-induced genes in G2M_checkpoint and E2F_Targets in the mutant uterus (Additional file 1: Fig. S4).

#### AF1 is necessary for progesterone to antagonize oestrogen action in OVX mice

We next evaluated the broad effect of AF1 mutations on progesterone-regulated gene expression. The Venn diagrams in Fig. 6B, C illustrate the number of differentially regulated genes between Ctrl and EB (red circle), between Ctrl and EBP (green circle), and between EB and EBP (blue circle) (padj < 0.05) in the WT and AF1_FFF mutant, respectively. A total of 9216 genes were significantly regulated by EB in the WT uterus and 8496 in the mutant. A total of 386 of the 9216 genes (4.2%) were differentially regulated between EBP and EB samples (Fig. 6B, the red oval, Additional file 8). These genes are significantly enriched with hallmark gene sets of Mtorc1_signaling, Interferon_gamma_response, and Hypoxia (Additional file 1: Fig. S5). In contrast, only 29 genes appear in AF1_FFF samples and 14 of the 29 genes are in the list of 386 genes (Fig. 6C). The heatmap of the 386 genes in Fig. 6D shows that approximately 80% of the genes (indicated by open brackets) were regulated by EB and EBP in the opposite direction in the WT uterus, indicating an antioestrogenic effect of progesterone. In contrast, these effects of progesterone were lost in the AF1_FFF uterus (Fig. 6D). The 31 genes in the blue oval also appear oppositely regulated by EB and EBP in the WT uterus (Fig. 6B, E, Additional file 9). These genes appear to be up- or downregulated by EB compared to Ctrl although the differences were not significant. Importantly, the antioestrogenic effect of progesterone on this group of genes was also abolished in the AF1_FFF uterus (Fig. 6C, E).

#### AF1 is required for synergistic repressive effect of progesterone and oestrogen on the uterus

The 1430 genes in the green oval were considered progesterone-regulated genes because these genes were significantly regulated by EBP but not by EB alone compared to the Ctrl (padj < 0.05) (Fig. 6B, Additional file 10). However, the heatmap of the 1430 genes suggests that most of the genes appear up- or downregulated by EB, albeit statistically not significant (Fig. 6F). The addition of progesterone results in significant up- or downregulation in the same direction as EB regulation, indicating synergistic effect between EB and progesterone. Although 850 genes were significantly regulated between EBP and Ctrl in the AF1 mutant (Fig. 6B), only 157 of the genes overlap with the 1430 progesterone-regulated genes in the WT. Thus, AF1 mutations affected ~ 89% of the EBP-regulated genes, and the mutations also gained about 700 significantly regulated genes, partly due to the secondary ripple effect. It is apparent that the downregulation by EBP was largely abolished in the AF1_FFF mutant (Fig. 6F). However, the upregulated genes were less affected by AF1 mutation in the heatmap.

Collectively, transcriptomic analysis of hormone-treated OVX mice revealed that progesterone exerts an antagonistic effect on approximately 5% of oestrogen-regulated genes. Progesterone also synergistically represses hundreds of genes with oestrogen although the effect of oestrogen alone was not significant. Importantly, AF1 is necessary for the antagonistic and synergistic repressive effect of progesterone on oestrogen-regulated genes.

### The activity AF1 on PGR-regulated genes in early gestation shows tissue selectivity

**Fig. 7:**
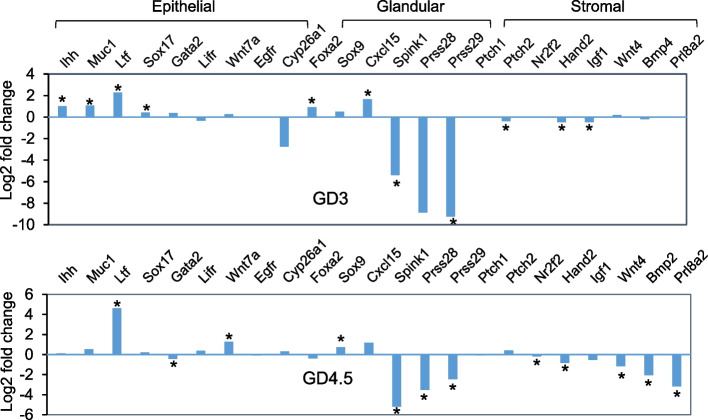
Tissue selective effect of AF1 mutations. Mutation-induced changes in the expression of progesterone-regulated genes on GD3 and GD4.5 are tissue-dependent. The epithelial, glandular, or stromal expression of the indicated genes is based on literature studies and may be developmental stage-specific. The log2 fold change is based on DESeq analysis of RNA-Seq data, *n* = 3 for GD3 and *n* = 4 for GD4.5 samples. Bars with an asterisk sign indicate significant change (padj < 0.05). Positive log2 fold changes indicate upregulation whereas negative log2 fold changes indicate downregulation in the mutant. Note that the large log2 fold value for *Cyp26a1* is due to one large outlier, and the change is not significant (padj = 0.11)

The intrinsically disordered domain has the propensity to interact with different proteins depending on the cellular environment, leading to different functional outcomes [24]. Since AF1 is intrinsically disordered, we evaluated whether AF1 mutations have any tissue-specific effect by examining the transcriptomic data for the expression of previously reported progesterone-regulated tissue-specific gene expression. *Ihh*, *Sox17*, *Gata2*, *Lifr*, *Wnt7a*, *Egfr*, and *Cyp26a1* are expressed primarily in the epithelium in early pregnancy and have been shown to contain PGR binding sites in ChIP assays [25–27]. Interestingly, all these genes except for *Gata2* were either upregulated or had no change in the mutant on GD3 and GD4.5 (Fig. 7). This suggests that AF1 is not required for PGR upregulation of these genes in epithelial cells. The downregulation of *Gata2* on GD4.5 can be explained by the understanding that *Gata2* is mainly expressed in epithelial cells before GD3.5 but also in stromal cells on GD4.5 [27]. *Foxa2*, *Sox9*, *Cxcl15*, *Spink1(Spink3)*, *Prss28*, and *Prss29* are glandular specific genes [28–33]. Interestingly, the effect of AF1 mutation on glandular-specific genes is not uniform. On the one hand, *Foxa2*, *Sox9*, and *Cxcl15* were either upregulated or did not change on GD3 and GD4.5. On the other hand, *Spink1(Spink3)*, *Prss28*, and *Prss29* were all markedly reduced on both days. This indicates the gene-specific activity of AF1 in glandular cells. The stromal genes, *Ptch1*, *Nf2f2*, *Fgf9*, *Wnt4*, *Bmp2,* and *Prl8a2* were not affected by AF1 mutation on GD3 except for *Ptch2* and *Hand2*, but most of them were significantly downregulated on GD4.5 in the mutant when stroma PGR is abundant (Fig. 7). As these stromal genes are important for uterine decidualization [34, 35], the observation provides a molecular explanation for the failure of decidualization in the AF1 mutant. Collectively, the data supports the widely presumed gene and tissue-specific activity of AF1.

### Evidence for PGR to interfere with ESR1-directed gene regulation at the genomic level

There are currently two intercalated models to explain how progesterone antagonize oestrogen action involving epithelium-stroma crosstalk. In one model, progesterone-induced Indian hedgehog (IHH) in the epithelium binds to PTCH1/2 in stromal cells to de-repress smoothened (SMO) G-protein coupled receptor, leading to upregulation of stromal NR2F2 (nuclear receptor subfamily 2, group F, member 2), also known as COUP transcription factor 2 (COUP-TFII) [36]. NR2F2 in stromal cells inhibits the activity of oestrogen receptor 1 (ESR1) [37], partly through inducing heart and neural crest derivatives expressed 2 (HAND2). In the second model, progesterone induces HAND2 in stromal cells, which inhibits the expression of members of the FGF family of growth factors. These FGF signal through FGFR→ERK1/2 in the epithelium to promote ESR1 phosphorylation and activation in the epithelium [38]. HAND2 is also reported to inhibit ESR1 activity through promoting proteasomal degradation of ESR1 [39]. The IHH and HAND2-induced signalling may operate in synergy or independently depending on the endometrial context.

**Fig. 8:**
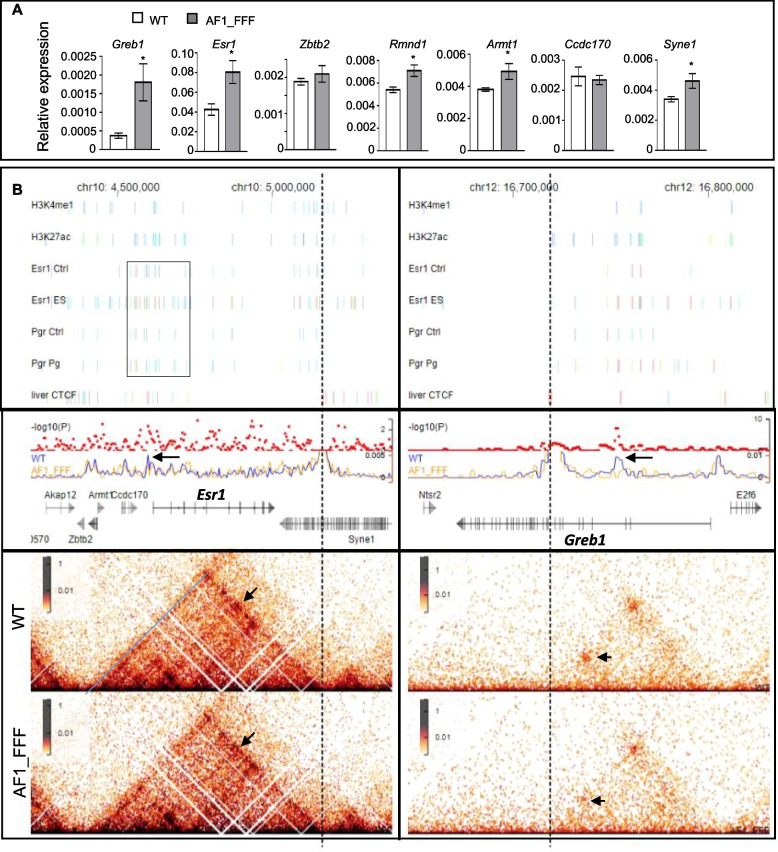
Evidence for the genomic mechanism of antagonism to ESR1 by PGR. **A** AF1 mutations caused upregulation of *Esr1*, *Greb1*, and some other genes (*Zbtb1*, *Rmnd1*, *Armt1*, *Cdc170*, and *Syne1*) in the ESR1-TAD. The results were obtained by qPCR of GD3 uterine samples and expressed as the relative change to housekeeping gene *36b4* (mean ± SEM, *n* = 6). **B** AF1 mutations alter the chromatin interactions in the *Esr1* (left side) and *Greb1* (right side) loci in the uterus. Top panel: composite illustration of intermingled ESR1 and PGR binding sites in the *Esr1* and *Greb1* gene loci in the uterus in the absence (Ctrl) or presence of oestrogen (ES) or progesterone (Pg). The enrichment of histone marks, H3K27ac, and H3K4me1 around the cluster of PGR and ESR1 binding sites indicates active enhancers. The CTCF binding data were from mouse liver. The chromatin binding data were obtained from the ChIP-Atlas (peak call *q* < 1E−10). The cluster of binding sites of ESR1 and PGR between the *Ccdc170* and *Esr1* genes correspond to the reported intergenic hormone control region (HCR) of *Esr1*. Different colours represent the significance of each peak (from low to high: blue, green, red). Bottom panel: Hi-C heatmaps of the WT and AF1 mutant in the *Esr1* and *Greb1* gene loci. The colour bar on the left of each matrix represents the signal intensity. The black arrow points to the loop of significant difference. Middle panel: virtual 4C signals at 5-kb and 2-kb resolution were obtained using sequence at 3 prime ends of the *Esr1* and *Greb1* domain, respectively (lower track); the corresponding hypergeomic *p*-values between the WT and AF1 mutant are shown above the 4C profile. The blue and orange lines of the virtual 4C plot represent WT and AF1 mutant, respectively. The black arrow points to the loop of significant difference

However, the hyper oestrogenic state and the delayed cessation of epithelial cell proliferation of the AF1_FFF uterus on GD3 are not associated with a corresponding decrease in the expression of the components in *Ihh* signalling (Fig. 7). Although *Nr2f2* is downregulated on GD3, its downstream effectors FGFs did not appear in the significantly dysregulated gene list (Additional file 2). The observations suggest that the antioestrogenic activity of PGR can be independent of IHH and HAND2 signalling. This is also supported by the evidence that antioestrogenic effect of progesterone in OVX mice is not associated with any upregulation of IHH or HAND2 signalling (Additional file 1: Fig. S6). On the other hand, we found significant upregulations of *Esr1* and *Greb1* in the mutant (Fig. 8A). This is consistent with the understanding that *Greb1* and *Esr1* expression are induced by oestrogen and inhibited by progesterone [40–43]. Since GREB1 is a significant ESR1 coactivator (Mohammed, D’Santos et al., 2013), their upregulations can conceivably enhance target gene expression in response to oestrogen.

We propose a direct genomic mechanism of antagonism to ESR1 activity in the uterus by PGR through inhibiting *Esr1* and *Greb1*. In breast cancer cells, PGR is known to regulate some target genes in opposite directions through intermingled chromatin binding with ESR1. Studies of topologically associated domains (TADs) in T47D cells reported the presence of ESR1-TAD that contains the *Esr1* gene and five other genes (*Zbtb2*, *Rnmd1*, *Armt1*, *Ccdc170*, and *Syne1*) on chromosome 6 [44]. Capture-C experiments and virtual 4C profiles showed that the promoters of these genes engage in frequent interactions with a 90-kb region known as hormone control region (HCR), which is defined as regulatory regions with clusters of ESR1 and PGR binding sites [45]. HCRs were found to establish long-distance interactions with gene promoters in the absence of or in response to hormones. Interestingly, *Esr1* and the five other genes in the mouse genome have identical linear arrangement on chromosome 10. qPCR analysis showed significant upregulations of *Esr1*, *Armt1*, *Rmnd1*, and *Syne1* in the AF1_FFF uterus on GD3 (Fig. 8A), suggesting that these genes were repressed by PGR through AF1. Data mining from the ChIP-Seq Atlas (https://chip-atlas.org/) showed that there is also a corresponding region enriched with intermingled ESR1 and PGR binding sites in uterine samples (Fig. 8B, top panel, the area in the rectangle) (Esr1_Ctrl, GSM894053; Esr1_Es, GSM894054; Pgr_Ctrl, GSM857545; Pgr_Pg, GSM857546) [25, 46]. This region is also enriched with histone marks H3K27ac and H3K4me1 for enhancers (H3K27ac, GSM3587124; H3K4me1, GSM3587115) (Fig. 8B). It is plausible therefore that in the GD3 uterus, PGR inhibits ESR1-induced expression of *Esr1* and *Greb1* directly at the genomic level.

This genomic mode of antagonism by PGR is supported by observations of altered chromatin interactions in *Esr1* and *Greb1* gene loci in the mutant. Hi-C libraries were generated from three uterine samples of each genotype and the data of each genotype were pooled together for analysis. Hi-C analysis of oestrogen-treated OVX adult uterus reported an interaction between the Igf1 super-enhancer region and the TSS in Hi-C analysis [47]. This was not observed in our study. Whole-genome loop and differential loop calling use Juicer’s HICCUPS. However, the differential loops called were statistically not significant between the two genotypes. We then zoomed in on chromatin interactions around known ESR1 target genes including *Esr1*, *Greb1*, *Igf1*, *Padi1*, *Foxa2*, and *Pgr* for the differences between the WT and the mutant, and interaction differences were identified in the *Esr1* and *Greb1* gene loci. The *Esr1* gene is wrapped in a hierarchical TAD containing loops, stripes, and subdomains (Fig. 8B, bottom panel), suggesting that the transcription of *Esr1* was regulated in the uterus through elaborate chromatin interactions. As there are several loops linked to the large domain’s 3 prime border, we use the 3 prime border as an anchor to check for the interaction differences between the wild-type and mutant samples by virtual 4C. A significant reduction in the interaction with the TSS region of the *Esr1* gene was observed in the mutant (Fig. 8B, middle panel). Both anchors of the loop contain PGR binding sites in the uterus and to the binding sites of CCCTC binding factors (CTCF) based on ChIP-Seq data from the liver (GSM2496432) (as the data are not available for the uterus) (Kentepozidou, Aitken et al., 2020) (Fig. 8B).

In contrast to *Esr1*, fewer loops were detected for the *Greb1* gene. In the WT samples, there were two prominent loops spanning the first half of the *Greb1* gene body that is enriched with ESR1 and PGR binding sites. These interactions were between the 1st and 9th introns and between the 3rd and 9th introns. Remarkably, the interaction between the 3rd and 9th introns was largely lost in the mutant. Virtual 4C analysis using the intron 9 region as an anchor confirmed loss of the loop in the mutant (Fig. 8B, middle panel, *p* < 0.000001). It is worth noting that the intron 3 region contains H3K4me1 and H3K27ac signals, supporting its enhancer activity and the regulatory role of the loop. Since the anchors of the lost loop also correspond to PGR and CTCF binding sites, the observation suggests that PGR represses *Esr1* and *Greb1* expression by cooperating with CTCF in an AF1-dependent manner.

## Discussion

The AF1 of nuclear receptors is known to play an important role in gene regulation, but its physiological significance in general is not clear. This study demonstrates that PGR AF1 is essential for progesterone to modulate embryo implantation and endometrial decidualization. AF1 mutant mice with triple mutations K461F_K478F_R489F are 100% infertile. The study also shed light on several functional features of AF1. First, the activity of AF1 on known PGR target genes is gene- and tissue-selective. AF1 mutations did not attenuate the expression of well-known PGR target genes in the luminal epithelium but markedly reduced the expression of glandular and stromal cell-specific genes on GD3 and GD4.5. Second, AF1 is important for progesterone to inhibit oestrogen-stimulated epithelial cell proliferation in early gestation (GD 2–3) and necessary for progesterone to stimulate uterine stromal cell proliferation. Third, progesterone antagonizes a small percentage (~ 5% depending on developmental stages) of oestrogen-regulated expression, and AF1 is essential for this antagonistic effect in both pregnancy and OVX mice models. Although AF1 is also required for the synergistic effect of progesterone with oestrogen, the effect seems more convincing on gene downregulation. Importantly, the study provides evidence for an alternative genomic mechanism of antagonism to ESR1 by PGR. It is found that the loss of the antioestrogenic activity of the AF1 mutant is not associated with decreased expression of the components in the canonical IHH-PTCH1/2-COUP-TFII pathway or in HAND2-FGFs signalling. Instead, upregulation of *Esr1* and *Greb1* in the mutant uterus is associated with reduced or loss of chromatin interactions in *Esr1* and *Greb1* gene loci. Interestingly, PGR and CTCF binding sites were found at the interaction anchors. We propose that the antioestrogenic effect of PGR at certain developmental stage (e.g. GD 2–3) is achieved by genomic upregulation of *Esr1* and its coactivator *Greb1* which in turn enhance the expression of other oestrogen target genes. Taken together, this study provides not only the first insight into the physiological role of AF1 but also suggests a genomic mechanism for progesterone to antagonize oestrogen action.

Since the upregulation of *Esr1* and *Greb1* in AF1 mutant is associated with reduced chromatin interactions in the specific locus, we postulate that these interactions are repressive to gene transcription and may involve CTCF which is important in regulating chromatin architecture and demarcating active and inactive chromatin domains [48]. It also acts as an insulator binding protein to block communications between enhancers and promoters. CTCF has been reported to repress oestrogen-induced gene transcription by hampering ESR1 chromatin binding and enhancer-promoter interaction in breast cancer cells [49]. As PGR is recruited to the CTCF binding region in ESR1-TAD in response to progesterone and inhibits gene expression [45], it could function through strengthening the repressive effect of CTCF. This is supported by the report that the binding of glucocorticoid receptor (GR) to the CTCF binding region promotes gene silencing of angiopoietin-like 4 (ANGPTL4) in hepatocytes [50]. The same mechanism can operate in the uterus because the anchors of the weakened interaction in *Esr1* and *Greb1* loci correspond to CTCF and PGR binding region. A ChIP-Seq analysis of the effect of AF1 mutation on PGR binding around these anchors would add confidence to this postulation.

It is worth noting that the proposed genomic mechanism of the antioestrogenic effect of PGR is likely different from how PGR regulates direct progesterone target genes. AF1 mutations caused a loss of the antioestrogenic effect of PGR on GD3 when PGR is primarily expressed in epithelia [10, 51]. This implies that the genomic antagonism occurs in the epithelium. However, AF1 mutations did not reduce the expression of progesterone target genes such as *Ihh*, *Muc1*, *Sox17*, *Wnt7a*, and *Ltfr* in the epithelia of the same sample on GD3, or on GD4.5. This suggests that AF1 is not critical for progesterone induction of its target genes in epithelia. Meanwhile, the expression of some of the glandular epithelium-specific genes is markedly reduced in the mutant, indicating the critical involvement of AF1 in this regulation. These different effects of mutations on epithelia suggest different modes of gene regulation by PGR AF1. We have reported stronger interactions of the AF1_FFF mutant with SRC-1 and with AF2 in coimmunoprecipitation and mammalian two hybrid assays [8]. The stronger interactions could effectively form a rigid tripartite structure that impairs the dynamics of the PGR transcription complex. This is supported by the Cryo-EM structure of human ESR1 that shows tripartite contacts among AF1, AF2, and SRC3 [52]. It is plausible that the AF1 facilitate dynamic interaction with coregulators and more rigid interaction of the AF1 mutant with coregulators and AF2 impairs its gene regulatory activity on progesterone-specific target genes, leading to gene downregulation in some tissues such as glandular epithelia and stromal cells.

## Conclusion

We report the first study of in vivo function of AF1 in the mouse uterus. We have provided strong evidence that AF1 essential for the antagonistic effect of progesterone on oestrogen action in the uterus. Notably, AF1 is necessary for progesterone to antagonize oestrogen-induced epithelial cell proliferation through inhibiting oestrogen-induced cell cycle genes. The study suggests a direct genomic mechanism of antagonism that is alternative to the prevailing mechanisms of stromal-epithelial crosstalk involving IHH or HAND2 signalling. AF1 mutations cause a significant reduction in chromatin interaction in selected loci corresponding to CTCF and PGR binding regions of the *Esr1* and *Greb1* genes. The genomic antagonism may operate in a specific developmental stage or in parallel with the paracrine mechanism to ensure reproductive success. Further studies are required to determine by ChIP-Seq whether AF1 mutations alter PGR binding to oestrogen target genes such as *Esr1* and *Greb1* and to progesterone-specific target genes. Given the importance of the antioestrogenic activity of progesterone in endometrial health, an in-depth understanding of how AF1 mediates the antioestrogenic effect is pivotal for the development of new therapeutic strategies for the clinical management of endometrial diseases. Additionally, mice with epithelial or stromal cell-specific AF1 mutations would be valuable for further characterization of the tissue-specific activity of AF1.

## Methods

### Ethical statement and mice are

C57BL/6J mice were housed in a specific pathogen-free (SPF) facility under a 12-h dark/light cycle and provided food and water ad libitum. All in vivo procedures and animal care were conducted in compliance with the Institutional Animal Care and Use Committee (IACUC) guidelines set by the National Advisory Committee for Laboratory Animal Research (NACLAR) of Singapore. All protocols of mice experimentation were approved by the Nanyang Technological University Institutional Animal Care and Use Committee (NTU-IACUC Protocol No: ARF SBS/NIE-A0304 and A18004). Generation of the mutant mouse model was carried out by The Animal Gene Editing Laboratory (AGEL), Biological Resource Centre (BRC), Agency for Science, Technology and Research (A*STAR) under IACUC protocol #151009 approved by A*STAR Institutional Animal Care and Use Committee (A*STAR-IACUC). The mouse line described here will be made available to the research community upon acceptance of the manuscript.

### Generation of PGR AF1_FFF mutant mice and genotyping

The PGR AF1 triple F mutant (461K→F, 478K→F, 489R→F) mouse line was generated by CRISPR/Cas9-mediated nucleotide mutagenesis. Two guide RNAs were designed to flank the mutant region in the first exon of the mouse PR gene. The sequences are gRNA-PR11: 5′ GGAGTGCATCCTGTACAAAG 3′ and gRNA-PR13: 5′ GCGGCCGGCAGGCTGTCCCG 3′. The mutations were introduced by a single-stranded oligonucleotide (OLN) containing the three mutations. The sequence of the 199 nucleotide OLN for FFF mutant is 5′AGCGCCGCGGTGTCGCCAGCGTCCTCCTCCGGCTCCGCGC TGGAGTGCATCCTGTACTTTGCGGAGGGCGCGCCGCCCACGCAGGGTTCGTTCGCGCCACTGCCGTGCTTTCCCCCAGCCGCCGGCTCCTGCCTACTACCCTTCGACAGCCTGCCGGCCGCCCCGGCCACCGCCGCAGCACCCGCCATCTACCAGCCGC 3′.

The gRNAs were synthesized by HiScribe™ T7 Quick High Yield RNA Synthesis Kit (NEB, #E2050), and Cas9 mRNA was synthesized by mMESSAGE mMACHINE T7 Ultra mRNA synthesis kit (Ambion, #AM1345). The pronuclei of one-cell embryos from C57BL/6J were injected with a mixture of two gRNAs, Cas9 mRNA, and the template OLN in 10 mM Tris.Cl, pH7.2 and implanted into pseudo-pregnant females. Founder animals were screened first by PCR with PR1/PR2 primers (5′ AGCCAGCTCCTCCACCTTCCCAGAC 3′ PR2 (reverse primer): 5′ AGGTAGTTAAGGTATGGCGGGTAGAC 3′). This is followed by restriction fragment length polymorphism (RFLP) of the PCR amplicons using Taq1. The targeted mutations in the positive founders were confirmed by sequencing. Founder animals containing the desired mutation were bred with the wild-type C57BL/6J mice to produce F1 heterozygotes. The F1 mutants were identified by PCR and confirmed by sequencing.

### Mice breeding and fertility assessment

Heterozygous to heterozygous mice breeding was initially strategized to assess the viability and fertility of either gender of the mutant. Crossing of heterozygous mice produced offspring with WT, heterozygous or homozygous in accordance with the Mendelian inheritance ratio of 1:2:1. However, homozygous AF1_FFF females failed to produce any pups after mating with heterozygous males for at least 6 months. On the other hand, homozygous males have normal fertility.

For the staging of pregnancy, the female and male mice were put together in the evening in two pairs per cage and were checked for copulation plug the next morning, when the presence of copulation plug was designated as gestation day (GD) 0.5. The mice in the late afternoon/early evening of the same day were defined to be on GD 1.

To determine the effect of the mutations on embryo implantation, anaesthetized female mice on GD4.5 were injected with 100 μl of 1% Evans Blue through the tail vein. Mice were sacrificed 3 min later, and the uterus was photographed for the staining of Evans blue at the embryo implantation sites. To evaluate the effect on decidualization, female mice were mated with vasectomized male mice to become pseudopregnant; 20 μl of sesame oil was injected into one uterine horn on GD 4 of pseudopregnancy to induce decidualization whilst the contralateral horn was served as a control with no sesame oil. The uterus was collected and photographed on day 8 of pseudopregnancy.

### Ki67 staining of the uterine tissue sections

The uterus was fixed in 4% paraformaldehyde and dehydrated through a series of graded ethanol followed by mixed xylenes prior to embedment in paraffin. Tissue sections of 5 μm thickness were stained with rabbit Ki67 antibody (Thermo Fisher Scientific, MA, USA) by the Advance Molecular Pathology Laboratory of the Institute of Molecular and Cell Biology, A*STAR, Singapore, using Bond™ Refine Detection Kit. Briefly, the antigen retrieval was done using Bond™ epitope retrieval solution. Three per cent hydrogen peroxidase was used to block endogenous peroxidase activity. Sections were blocked with 10% goat serum for 30 min before incubation with Ki67 antibody at 1:100 dilution. The polymeric horseradish peroxidase (HRP)-linker antibody conjugate was incubated on sections for 5 min, and the Ki67 signals were detected with Bond™ mixed DAB Refine solution. The sections were counterstained with haematoxylin before dehydration and mounting with synthetic mounting medium.

### Determination of serum concentration of progesterone on GD3 and GD4.5

The sera in GD3 and GD 4.5 mice were collected by cardiac puncture under anaesthesia with a 20G needle. Blood collected was left at room temperature for up to 30 min before centrifugation at 10,000 rpm at 4 °C for 10 min. The translucent fluid at the top was snap-frozen in liquid N2 and kept at − 80 °C for further analysis.

Progesterone concentration in the serum was determined by PGR reporter gene assay. Hela cells were seeded in 60-mm dishes at 4.0 × 10^5^ each in phenol-red-free DMEM containing 5% DCC-FCS and 2mM l-glutamine. Twenty-four hours post seeding, cells were transfected with plasmids (5 ng pcDNA3.1-PGRB, 1 μg PRE-2X-TATA-LUC, and 1 ng Renilla Vector pRL-CMV (generously provided by Dr. M.-J. Tsai, Baylor College of Medicine, Houston, TX) using polyethylenimine (PEI) (Sigma, P3143) per manufacturer’s protocol. Mouse serum collected from GD3 and GD 4.5 was added to the culture plates at 24 h after transfection and incubated for 16 h. Cells were then collected and lysed with 1× lysis buffer from the Dual-Luciferase® Reporter System (Promega, E1960). Lysate was collected and analysed with a computer-controlled microplate luminometer (GloMax® Multi+ Microplate Multimode Reader with Instinct®, Promega). Activities of Renilla and Firefly luciferase were measured and recorded as per manufacturer’s protocol. Progesterone concentration in serum samples was calculated against a standard curve obtained in the same luciferase assay with known progesterone concentrations (0 nM, 0.2 nM, 0.5 nM, and 1 nM for GD3 samples, and 0 nM, 0.1 nM, 0.2 nM, 0.5 nM, 1 nM, 2 nM, 5 nM, and 10 nM for GD4.5 samples). Serum progesterone concentrations were calculated as ng/ml.

### Hormone treatment for RNA-Seq analysis

To evaluate the effect of AF1 mutations on progesterone-regulated gene expression in the uterus, mice were ovariectomized at 8–9 weeks old to remove the endogenous source of oestrogen and progesterone. Two weeks after the ovariectomy, the WT or AF1_FFF mice were randomly divided into 3 groups. The control (Ctrl) group was injected with sesame oil subcutaneously daily for 2 days. The second group was injected with 17β-estradiol benzoate (EB group) (20 μg/kg) daily for 2 days. The third group was given EB injection on the first day and progesterone injection at 20 mg/kg 24 h later (EBP group). Forty-eight hours after the first injection, the uterus was collected and snap-frozen for RNA collection and RNA-Seq analysis.

### RNA-Seq analysis of gene expression

RNA-Seq analysis was conducted to determine the effect of AF1 mutations on global gene expression in mice in early pregnancy and in ovariectomized mice treated with Ctrl, EB, or EBP. Total uterine RNA was extracted using TRIzol Reagent (Thermo Fisher Scientific, USA) and treated with DNase I using the DNA-free™ DNA Removal Kit (Invitrogen, Carlsbad, CA, USA) to remove contaminating DNA. The total RNA was processed for library preparation and paired-end sequencing by the Genome Institute of Singapore, Agency for Science, Technology and Research) on Illumina HiSeq4000. Read quality was evaluated using FastQC [53]. After quality control, the transcript expression was quantified against the Ensembl mm10 cDNA library using Salmon v1.1.0 [54]. Transcript counts were summarized to gene counts using tximport [55]. Differential genes were identified using DESeq2 [56] with the following thresholds: |log2fc| ≥ 1 and Benjamini-Hochberg corrected *p*-value (padj) < 0.05. Heatmaps were generated using the pheatmap package that was run using RStudio [57, 58]. Venn diagram was generated using the online program Venny [59].

### The gene set enrichment analysis (GSEA)

The GSEA was performed using the software downloaded from https://www.gsea-msigdb.org/gsea/index.jsp [60, 61]. The analysis was conducted with the default parameters, except for “permutation type” where “gene_set” was used. Enriched terms with a false discovery rate (FDR) of *q* < 0.25 was considered statistically significant. Hallmark gene sets from the Molecular Signature Database (MSigDB) describe the coordinated expression of defined biological processes [62]. Output from C3 all transcription factor targets (TFT) analysis indicate transcription factors as an upstream regulator.

### Hi-C analysis

Differences in chromatin interactions between the WT and AF1_FFF uterus were analysed by Hi-C using Arima-HiC+ kit (Arima Genomics, San Diego) according to the manufacturer’s protocol with minor modifications. Briefly, three WT and three AF1_FFF uterine samples obtained on GD3 were pulverized in liquid N2, cross-linked and lysed. The samples were digested using a restriction enzyme cocktail and the 5′-overhangs were biotin labelled. The DNA were ligated, fragmented, and collected by biotin pull down. The Hi-C libraries were prepared using the Kapa HyperPrep Library Kit (Roche) and sequenced in a NovaSeq 6000 (Illumina) by 2 × 150 bp mode.

Paired-end sequencing reads of Hi-C experiment were aligned to mm 10 genome and processed into interaction matrices by using distiller-nf pipeline (aligner: bwa1; alignment processor: pairtools; matrix builder: cooler). Hi-C loops and differential loops were detected at 2.5 and 10 kb resolution by using HiCCUPS and HiCCUPSdiff of Juicer tools. The parameters are: -r 2000,5000,10000 -f 0.05,0.1,0.15 -p 6,4,2 -i 14,10,6 -d 20000,20000,21000. Higlass3 was used to visualize Hi-C data. For the virtual 4C plot, we selected corresponding row of the anchor of interest from the Hi-C raw matrix and then normalized it. A hypergeometric test was conducted for each bin by using the raw count in the 4C plot to check the significance of the differences.

### Western blotting analysis of PGR protein

Ovariectomized mice were treated with EB at 20 μg/kg for 24 h before snapping frozen in liquid N2. The uterine tissues were pulverized with a mortar and pestle that were pre-cooled in liquid N2. Total protein was extracted by a mechanical homogenizer in cold lysis buffer (50 mM HEPES, pH 7.5, 150 mM sodium chloride, 5 μg/ml pepstatin A, 5 μg/ml leupeptin, 2 μg/ml aprotinin, 1 mM PMSF, 100 mM sodium fluoride, and 1 mM sodium vanadate and 1% Triton X-100). The concentration of protein lysates was determined using Pierce BCA Protein Assay Kit (Thermo Fisher Scientific, MA, USA). Fifty micrograms of total protein of each sample were resolved by SDS-PAGE (sodium dodecyl sulphate–polyacrylamide gel electrophoresis) and transferred onto nitrocellulose membrane. The membrane was blocked with 2.5% bovine serum albumin and probed with PGR antibody clone H190 (Santa Cruz Biotechnology, Dallas, USA) and secondary antibody conjugated with horseradish peroxidase that was detected by Immobilon Western Chemiluminescent HRP substrate (Merck Millipore, Billerica, MA, USA) and X-ray film.

### Real-time quantitative PCR (qPCR) analysis

Total RNA was reverse transcribed using qScript cDNA SuperMix (Quantabio). Real-time qPCR was carried out with KAPA SYBR FAST qPCR reagent (KAPA Biosystems, Massachusetts, USA) on the Quantstudio 6 Flex Real-Time PCR System (Applied Biosystems, MA, USA). qPCR for each primer set was conducted in duplicates. For quantitative analysis, the threshold cycle (*C*_t_) of the gene of interest was normalized to *C*_t_ value of *36b4* in the same sample. The relative quantification was made using the 2^−Δ*C*t^ method [63]. The relative gene expression is expressed relative to the housekeeping gene. Primers are listed in Additional file 11.

### Statistical analysis

When comparing between the WT and AF1_FFF, statistical significance was determined using a two-tailed unpaired Student’s two-tailed *t*-test. All statistical analysis was performed using the GraphPad Prism 7 software. *p*-value: < 0.05 (*), < 0.01 (**), < 0.001 (***), and < 0.0001 (****).

## Supplementary Information

**Additional file 1: Fig. S1.** Genotyping, PGR levels fertility of AF1 mutant mice. **Fig. S2.** AF1_FFF mutation did not affect nuclear localization of PGR. **Fig. S3.** Serum concentrations of progesterone on GD3 and GD4.5 between the genotypes are not significantly different. **Fig. S4.** AF1 mediates progesterone suppression of estrogen regulated cell proliferation genes. **Fig. S5.** AF1 mediates progesterone suppression of estrogen regulated Mtorc1 signaling, interferon gamma response, and hypoxia Hallmark gene sets. **Fig. S6**. The antiestrogenic effect of progesterone in the uterus of OVX mice is independent of IHH and HAND2 signaling.

**Additional file 2.** 1261 Differentially expressed genes between the WT and AF1_FFF uterus on GD3.

**Additional file 3.** 807 Common genes between estrogen-regulated in the OVX WT and dysregulated in the AF_FFF GD3.

**Additional file 4.** 1341 Differentially expressed genes between the WT and AF1_FFF uterus on GD4.5.

**Additional file 5.** 901 Common genes between estrogen-regulated in the OVX WT and dysregulated in the AF_FFF GD4.5.

**Additional file 6.** Common and unique EB regulated genes on GD 3 and GD 4.5.

**Additional file 7.** G2M_checkpoint and E2F_Targets are not in the Top 20 gene sets enriched in EBP group vs Ctrl.

**Additional file 8.** 31 genes significantly regulated between EBP and EB.

**Additional file 9.** 386 EB-regulated genes also regulated by progesterone compared between EB and EBP.

**Additional file 10.** 1430 genes that are significantly regulated between EBP and Ctrl but not between EB and Ctrl.

**Additional file 11.** qPCR Primer list.

## Data Availability

The AF1_FFF mice with the strain name MMRRC: [67160-JAX], C57BL/6 J-Pgrem2Lvntu/Mmjax are available from The Jackson Laboratory. The Hi-C sequencing data are available from http://www.ncbi.nlm.nih.gov/bioproject/873212 with a BioProject ID of PRJNA873212.
