# Author Correction: Activation function 1 of progesterone receptor is required for progesterone antagonism of oestrogen action in the uterus

**DOI:** 10.1186/s12915-023-01543-z

**Published:** 2023-02-16

**Authors:** Shi Hao Lee, Chew Leng Lim, Wei Shen, Samuel Ming Xuan Tan, Amanda Rui En Woo, Yeannie H. Y. Yap, Caitlyn Ang Su Sian, Wilson Wen Bin Goh, Wei-Ping Yu, Li Li, Valerie C. L. Lin

**Affiliations:** 1 School of Biological Sciences, Nanyang Technological University, Singapore, 637551 Singapore; 2 College of Informatics, Huazhong Agricultural University, Wuhan, China; 3 Present Address: Department of Oral Biology and Biomedical Sciences, Faculty of Dentistry, MAHSA University, Bandar Saujana Putra, 42610 Jenjarom, Selangor Malaysia; 4 Animal Gene Editing Laboratory (AGEL), Biological Resource Centre, Agency for Science, Technology and Research (A*STAR), 61 Biopolis Drive, Proteos, Singapore, 138673 Singapore; 5 Institute of Molecular and Cell Biology, Agency for Science, Technology and Research (A*STAR), 61 Biopolis Drive, Proteos, Singapore, 138673 Singapore

**Author Correction: BMC Biol 20, 222 (2022)**

**https://doi.org/10.1186/s12915-022-01410-3**

The original article [1] contains two incorrect terms in the following sentence:

“We propose that the antioestrogenic effect of PGR at certain developmental stage (e.g. GD 2–3) is achieved by genomic upregulation of Esr1 and its coactivator Greb1 which in turn enhance the expression of other oestrogen target genes."

In the aforementioned sentence, 'upregulation' should instead be 'downregulation', and 'enhance' should instead be 'reduce', yielding the following, correct sentence:

“We propose that the antioestrogenic effect of PGR at certain developmental stage (e.g. GD 2–3) is achieved by genomic downregulation of Esr1 and its coactivator Greb1 which in turn reduce the expression of other oestrogen target genes."
