# Supplementary material for: Activation function 1 of progesterone receptor is required for progesterone antagonism of oestrogen action in the uterus
Source: BMC Biol. 2022 Oct 5;20:222. doi: 10.1186/s12915-022-01410-3 (PMC9535881; doi:10.1186/s12915-022-01410-3)
Supplement: Supplementary file 1 — Additional file 1: Fig. S1. Genotyping, PGR levels fertility of AF1 mutant mice. Fig. S2. AF1_FFF mutation did not affect nuclear localization of PGR. Fig. S3. Serum concentrations of progesterone on GD3 and GD4.5 between the genotypes are not significantly different. Fig. S4. AF1 mediates progesterone suppression of estrogen regulated cell proliferation genes. Fig. S5. AF1 mediates progesterone suppression of estrogen regulated Mtorc1 signaling, interferon gamma response, and hypoxia Hallmark gene sets. Fig. S6. The antiestrogenic effect of progesterone in the uterus of OVX mice is independent of IHH and HAND2 signaling. [file 12915_2022_1410_MOESM1_ESM.pdf]

Supplementary Figures

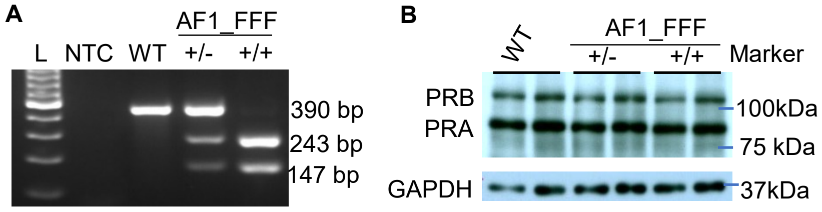

C

AF1_FFF mice are infertile			
Male	Female	Sample	Litter Size
Wt/FFF	Wt/FFF	n=10	8.9± 0.48
FFF/FFF	FFF/FFF	n=10	0 ± 0.0
Wt/Wt	FFF/FFF	n=10	0 ± 0.0

Supplementary Figure 1. Genotyping, PGR levels fertility of AF1 mutant mice. A. Genotyping of AF1_FFF mice. PCR of genomic DNA was carried out using PR1 (forward primer): 5' AGCCAGCTCCTCCACCTTCCCAGAC 3' And PR2 (reverse primer): 5' AGGTAGTTAAGGTATGGCGGGTAGAC 3'. The thermal cycle condition is the following: 95oC 2 min, 95oC 30 sec, 55oC 30 sec, 72oC 1 min (x35 cycles), 72oC 10 min, 10oC hold. The PCR product was digested with Taq1 orTaq.1$^{\alpha}$ at 65oC for 1.5 hour. The digestion products were resolved on 2% agarose gel. The WT DNA yields a 390 bp product and the mutant will have a 243 bp and a 147 bp band. **B.** The protein levels of PGR isoform A and B are similar in the WT and the AF1_FFF mutant uterus. OVX mice of each genotype were treated with 20 µg EB/kg body weight for 24h before the whole uterus were collected for Western blotting analysis. GAPDH is probed as loading control. **C**. The heterozygous female mice breed normally and the homozygous AF1_FFF mice are 100% infertile.

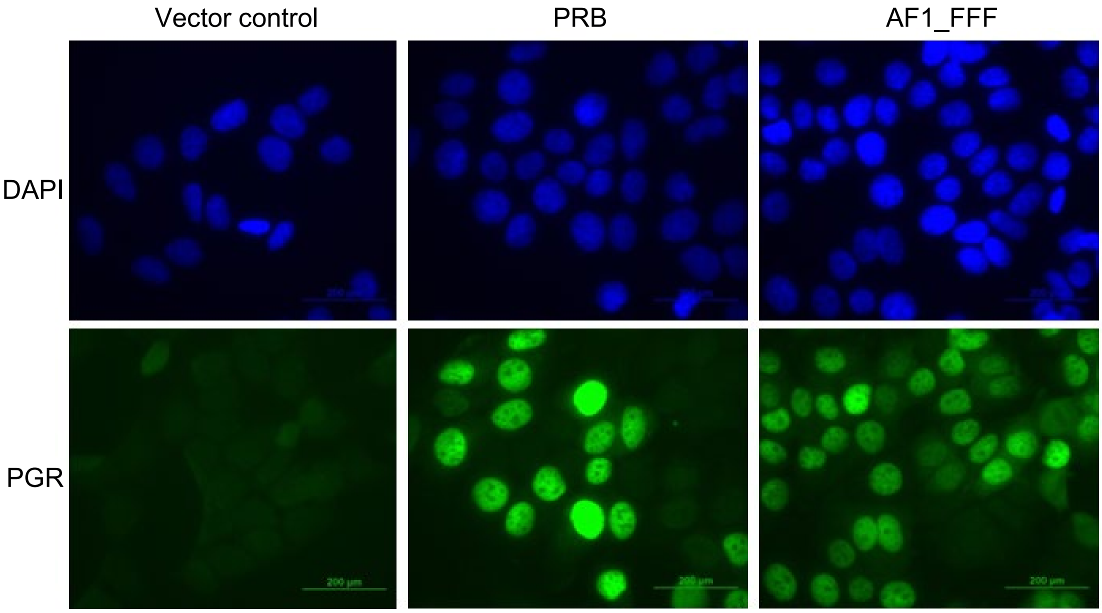

Supplementary Figure 2 AF1_FFF mutation did not affect nuclear localization of PGR.
Cells were transduced with empty lentiviral vector, vector containing WT or AF1_FFF PGRB cDNA. PGR is probed PGR antibody H-190 from Santa Cruz Biotechnology followed by incubating with fluorescein-conjugated secondary antibody. DAPI was used as a nuclear counterstain.

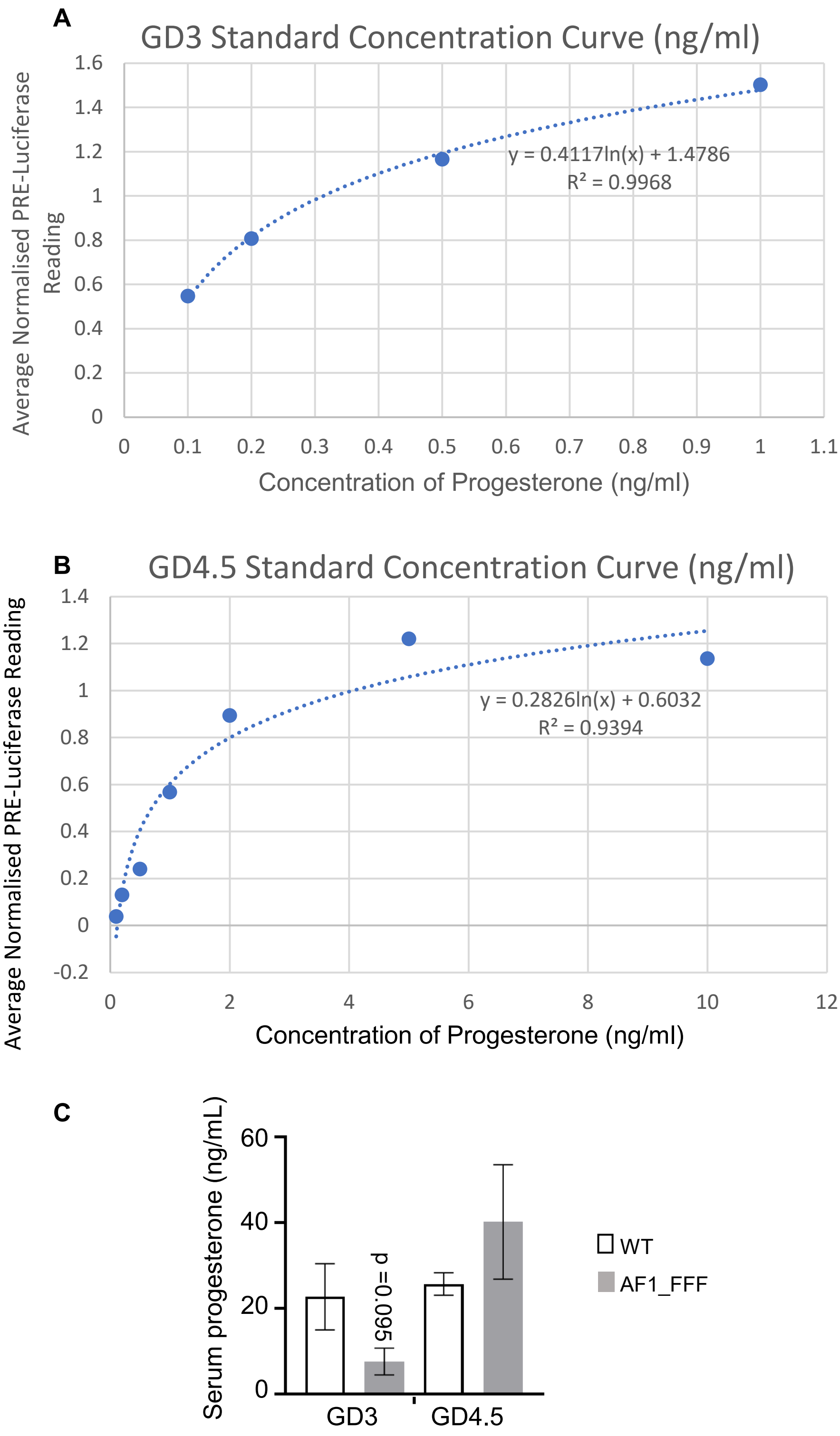

Supplementary Figure 3. Serum concentrations of progesterone on GD3 and GD4.5 between the genotypes are not significantly different. Progesterone concentrations in sera were analyzed by PGR reporter gene assay against progesterone standards in the standard curve. The PRE-2X-TATA-Luc vector was used as the reporter plasmid and pRL-Renilla Vector was used as transfection control. **A** and **B**, the normalized luciferase readings from progesterone standard concentrations was used to construct the standard curve for GD3 and GD4.5, respectively. **C**, Serum concentration of progesterone in GD3 and GD4.5 mice. The relative luciferase reading of each sample was used to calculate the concentration based on the equation derived from the standard curve. . The results are expressed as mean±SEM. N=7 for GD3 and n=4 for GD4.5 samples. Protocol details are in the main manuscript.

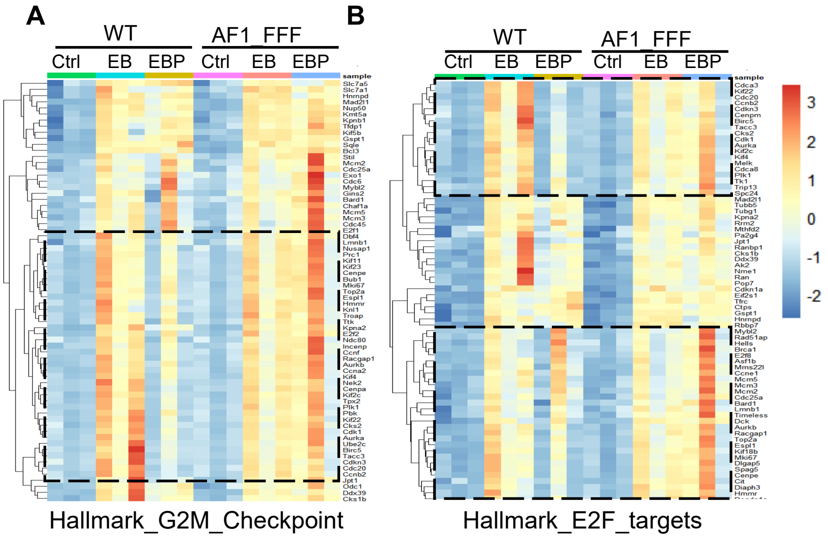

Supplementary Figure 4. AF1 mediates progesterone suppression of estrogen regulated cell proliferation genes. Progesterone antagonizes EB induced G2M (**A**) checkpoint and E2F (**B**) targets gene sets in a AF1-dependent manner in OVX mice. The heatmap was based on core enriched genes positively regulated by EB in these gene sets. The EB upregulated genes enclosed in dotted rectangle were downregulated by progesterone in the WT and the effect was attenuated in the AF1_FFF uterus.

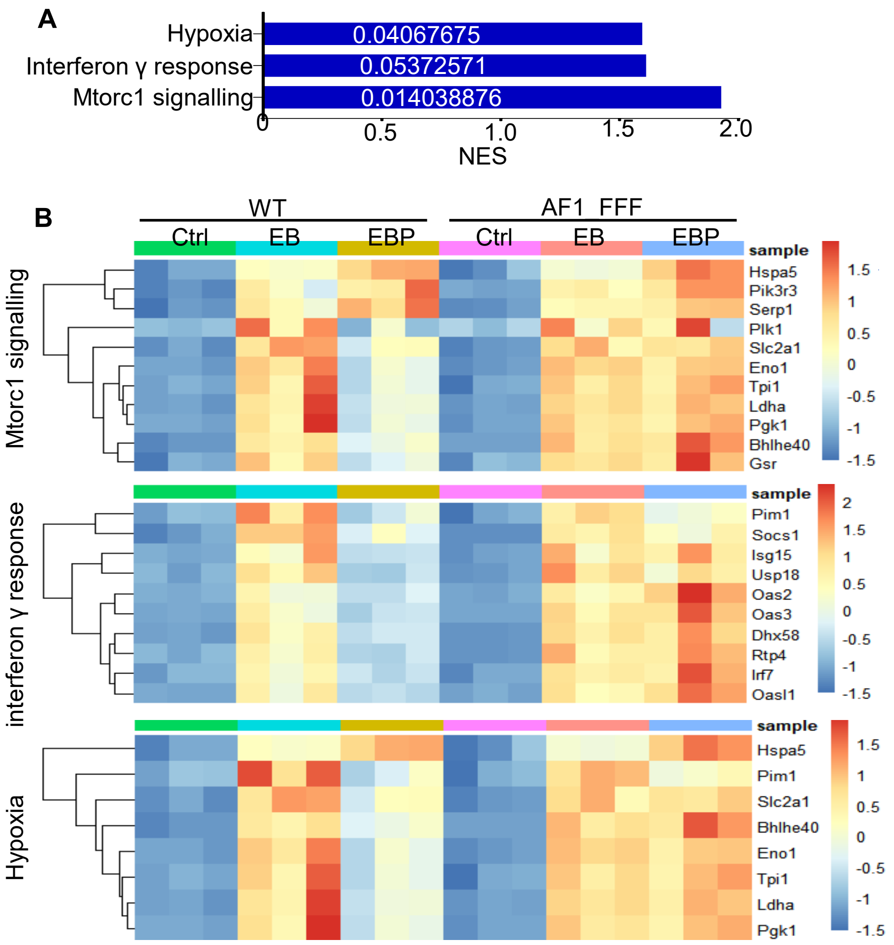

Supplementary Figure 5. AF1 mediates progesterone suppression of estrogen regulated Mtorc1 signalling, interferon gamma response, and hypoxia Hallmark gene sets. **A**. NES and FDR q values of the three Hallmark gene sets. The numbers in the blue bars are FDR q values. **B**. Heatmaps of the core enriched genes of the 3 Hallmark gene sets.

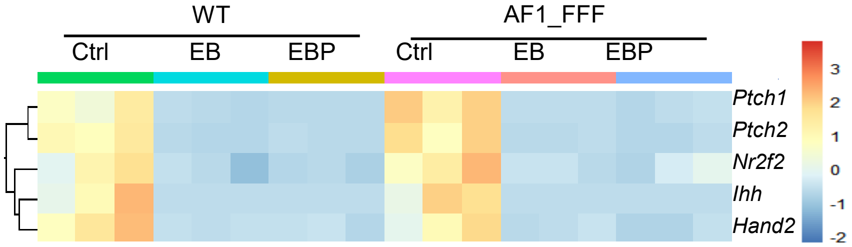

Supplementary Figure 6. The antiestrogenic effect of progesterone in the uterus of OVX mice is independent of IHH and HAND2 signaling. Estrogen inhibited the expression *Ptch 1, Ptch2, Nr2f2*, *Ihh* and *Hand2* in OVX mice, and the addition of progesterone or AF1 mutation had no effect. The heatmap was generated with transcript counts of individual genes from RNA-Seq data (n=3).
